# Extraction of Astaxanthin from *Haematococcus pluvialis* and Preparation of Astaxanthin Liposomes

**DOI:** 10.3390/molecules29143320

**Published:** 2024-07-15

**Authors:** Fei Luo, Shuai Wang, Xuwu Zhang, Zhiwei Liu, Ruiyan Zhu, Weili Xue

**Affiliations:** 1 Hebei Key Laboratory of Nano Biotechnology, College of Environmental and Chemical Engineering, Yanshan University, Qinhuangdao 066004, China; luofei0502@163.com (F.L.); ysdxws123@163.com (S.W.); xwzhang@ysu.edu.cn (X.Z.); zwliu@ysu.edu.cn (Z.L.); 2 COFCO Huaxia Great Wall Wine Co., Ltd., Changli 066600, China

**Keywords:** *Haematococcus pluvialis*, astaxanthin extraction, astaxanthin liposomes, enhanced stability, antioxidant activity

## Abstract

Astaxanthin has 550 times more antioxidant activity than vitamin E, so it can scavenge free radicals in vivo and improve body immunity. However, the poor stability of astaxanthin becomes a bottleneck problem that limits its application. Herein, *Haematococcus pluvialis* (*H. pluvialis*) as a raw material was used to extract astaxanthin, and the optimal extraction conditions included the extraction solvent (EA:EtOH = 1:6, *v*/*v*), extraction temperature (60 °C), and extraction time (70 min). The extracted astaxanthin was then loaded using lecithin to form corresponding liposomes via the ethanol injection method. The results showed that the particle size and zeta potential of the prepared liposomes were 105.8 ± 1.2 nm and −38.0 ± 1.7 mV, respectively, and the encapsulation efficiency of astaxanthin in liposomes was 88.83%. More importantly, the stability of astaxanthin was significantly improved after being embedded in the prepared liposomes.

## 1. Introduction

Astaxanthin ((3S,3′S)-3,3′-Dihydroxy-β, β-carotene-4,4′-dione, C_40_H_52_O_4_) is a secondary carotenoid with super-antioxidant activity (1000 times greater than vitamin E), which endows astaxanthin with outstanding physiological functions such as free radical and reactive oxygen species scavenging [1]. Therefore, astaxanthin can be widely used in biomedical fields such as protecting the optic nerve and central nervous system, preventing ultraviolet radiation and cardiovascular diseases, enhancing immunity and energy metabolism, relieving exercise fatigue, resisting inflammation and infection, and inhibiting tumors and diabetes [2,3,4]. In addition, it also can be used in the breeding of aquatic animals as well as in cosmetics and advanced nutrition and health products [5]. However, its application is extremely restricted due to its poor stability, poor water solubility, and low bioavailability.

At present, astaxanthin is mainly produced by chemical synthesis and biological acquisition [6]. Although synthetic astaxanthin has relatively high purity, it is expensive to produce and the racemic property of the product is difficult to control. In addition, the absorption efficacy is low compared with natural astaxanthin [7]. *Haematococcus pluvialis* (*H. pluvialis*) can rapidly synthesize and accumulate astaxanthin under conditions of adversity and stress such as strong light, high temperatures, nutrient (nitrogen and phosphorus) starvation, and high salt conditions. The accumulation amount of astaxanthin produced by *H. pluvialis* can be up to 4.0% of the dry weight of algae cells, which is much higher than that extracted from shrimp and crab shells and that produced by *Phaffia rhodozyma* fermentation (0.15~0.4%), so *H. pluvialis* is recognized as the ideal source of natural astaxanthin [8,9,10].

Liposome microcapsules, types of bilayer vesicles with cell-membrane-like structures, are composed of natural phospholipids or synthetic phospholipids and other stabilizing components (such as cholesterol) [11]. As a bioactive substance or drug-loading carrier, liposomes have the following merits. They can effectively improve the long-term stability of active ingredients and prevent the chemical and biological degradation of loaded drugs during the delivery process [12]. The water solubility of the loaded drugs can be improved and the nonspecific side-effects and toxicity of encapsulated drugs can be decreased, accompanied by augmented therapeutic efficacy and increased bioavailability [13]. The liposome surface can be modified using a variety of special molecules or functional groups (such as polyethylene glycol, chitosan, and target groups) to provide versatility so that bioavailability, biocompatibility, and sustained release can be achieved [14,15].

In this study, *H. pluvialis* as a raw material was used to extract astaxanthin via the solvent extraction method. According to the single-factor experiment, the influencing factors on the extraction amount of astaxanthin were investigated, including the type of extraction solvent, the extraction time and temperature, the solid–liquid ratio, and the extraction times. The extracted astaxanthin, a type of liposoluble component, was then encapsulated using lecithin to prepare astaxanthin liposomes via the ethanol injection method. Finally, the physicochemical properties and bioactivities were characterized and investigated.

## 2. Results and Discussion

### 2.1. Standard Curve of Astaxanthin

**Figure 1:**
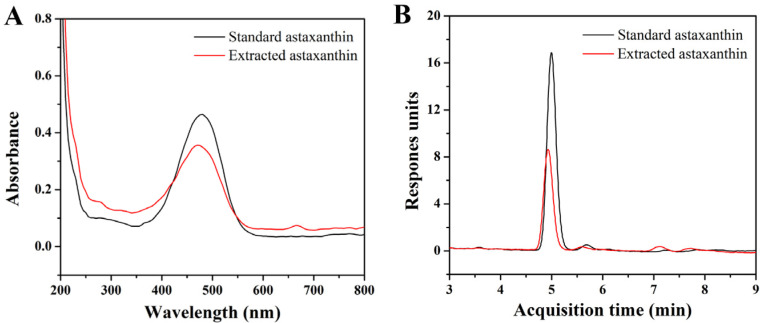
(**A**) UV–vis absorption spectra of standard astaxanthin and extracted astaxanthin samples; (**B**) LC-MS spectra of standard astaxanthin and extracted astaxanthin samples (columns: Agilent XDB-C18; mobile phase: H_2_O/MeOH; flow speed: 0.8 mL/min; injection amount and concentration: 20 μL and 4 μg/mL).

From Figure 1A, it can be seen that the maximum absorption wavelength of astaxanthin was 479 nm, which was then selected to determine the standard curve of the standard astaxanthin sample. The result indicated that the concentration of astaxanthin had a linear relationship with the absorbance within the range of 1~5 μg/mL. The regression equation of the standard curve was y = 0.049 + 0.105x, where x represented the astaxanthin concentration and y represented the corresponding absorbance. The coefficient of determination (R^2^) was 0.9958, indicating a good linearity and qualification for the following content measurement of astaxanthin. The LC-MS spectra results (Figure 1B) indicated that the purities of the standard astaxanthin sample and extracted astaxanthin sample were 95.99% and 89.78%, respectively.

### 2.2. Single-Factor Experimental Results

**Figure 2:**
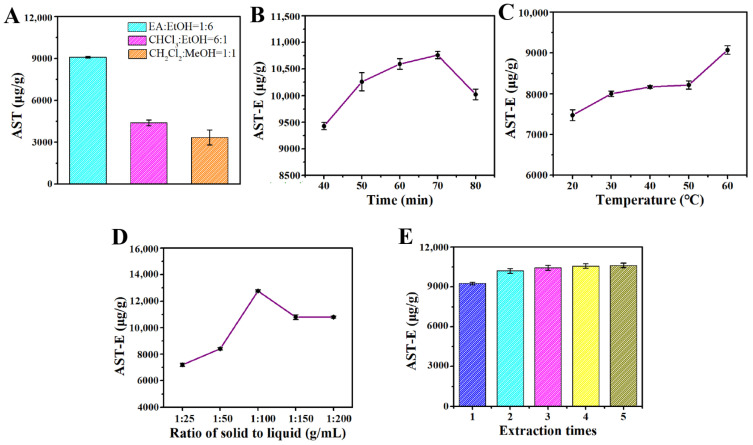
(**A**) Effect of extraction types on astaxanthin extraction amount; (**B**) effect of extraction time on astaxanthin extraction; (**C**) effect of extraction temperature on astaxanthin extraction; (**D**) effect of solid–liquid ratio on astaxanthin extraction; (**E**) effect of extraction times on astaxanthin yield. All data are shown as the mean ± SD; *n* = 3.

According to Figure 2A, the extraction effects of the different extraction solvents on the amount of astaxanthin extracted from *H. pluvialis* were significantly different. The extraction solvent containing ethyl acetate (EA) and ethanol (1:6, *v*/*v*) had the best extraction effect, and the extraction amount of astaxanthin was 9095.238 μg/g. Therefore, the extraction solvent containing EA and ethanol with a volume ratio of 1:6 was selected as the optimal extraction solvent for subsequent use.

The extraction time had an influence on the extraction amount of astaxanthin from *H. pluvialis* to some degree. According to Figure 2B, for extraction times from 40 to 70 min, the extraction amount of astaxanthin showed an upward trend. When the extraction time was 70 min, the extraction amount of astaxanthin reached the maximum value of 10,761.905 μg/g. With a prolonged time, the extraction amount of astaxanthin began to rapidly decrease. Therefore, the optimal extraction time was 70 min in this study.

The extraction temperature had an influence not only on the extraction amount of astaxanthin, but also on the stability of the extracted astaxanthin. Astaxanthin is inactivated when the temperature is higher than 60 °C [16]. From Figure 2C, it can be seen that the extraction amount of astaxanthin increased when the extraction temperature elevated from 20 °C to 60 °C. The extraction amount of astaxanthin reached the maximum value of 9095.238 μg/g when the extraction temperature was 60 °C, so the optimal extraction temperature was 60 °C.

In industrial production, the larger the solid–liquid ratio, the higher the requirements for the impurity removal process and distillation process. The purification time will also increase, relatively. The purpose of this experiment was to screen the relatively low solid–liquid level with the relatively high extraction amount of astaxanthin. Figure 2D shows that the extraction amount of astaxanthin first increased and then decreased with an increase in the solid–liquid ratio, and the extraction amount of astaxanthin reached the maximum value of 12,771.429 μg/g when the solid–liquid ratio was 1:100 (g/mL). Therefore, the optimal solid–liquid ratio was 1:100 (g/mL).

Although the extraction amount of astaxanthin increased with an increase in the extraction times, in industrial production, a longer extraction time denotes a greater production time, an increase in the solvent used, and a relatively increased purification time. Therefore, a relatively higher extraction amount with a lower extraction time is the optimal production process. The result from Figure 2E shows that the cumulative extraction amount of astaxanthin increased with an increase in the extraction time. After two extraction times, the increment of extracted astaxanthin became very low. Therefore, two extraction times were selected as the optimal extraction times, taking the extraction amount and energy consumption into account.

### 2.3. Characterization Results of Astaxanthin Liposomes

#### 2.3.1. Morphology of Astaxanthin Liposomes

**Figure 3:**
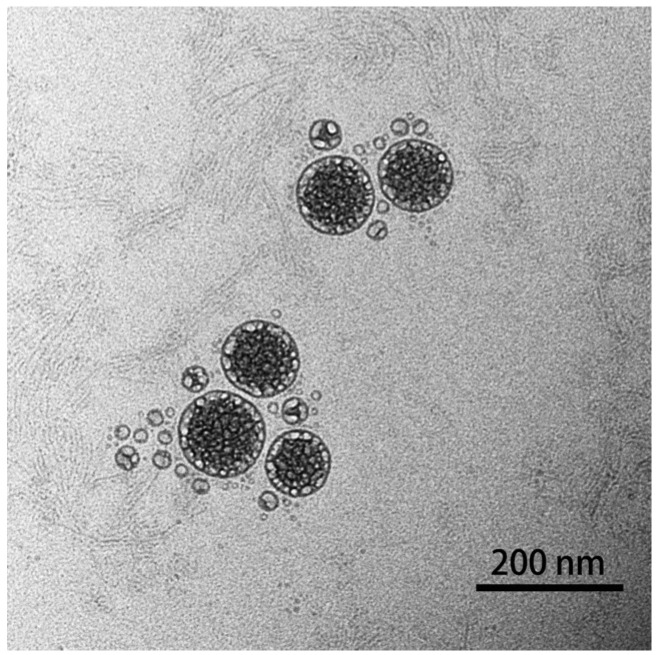
TEM image of prepared astaxanthin liposomes.

The morphology of the prepared nanoparticles was observed using TEM (Figure 3), and the result showed that astaxanthin liposomes had regular spherical shapes, a smooth surface, a uniform particle size, and no aggregation phenomena, indicating the good stability of the system [17]. In addition, the result also demonstrated that the astaxanthin liposomes prepared using the ethanol injection method in this experiment had good monodispersity.

#### 2.3.2. Particle Size Distribution and Zeta Potential of the Prepared Astaxanthin Liposomes

**Figure 4:**
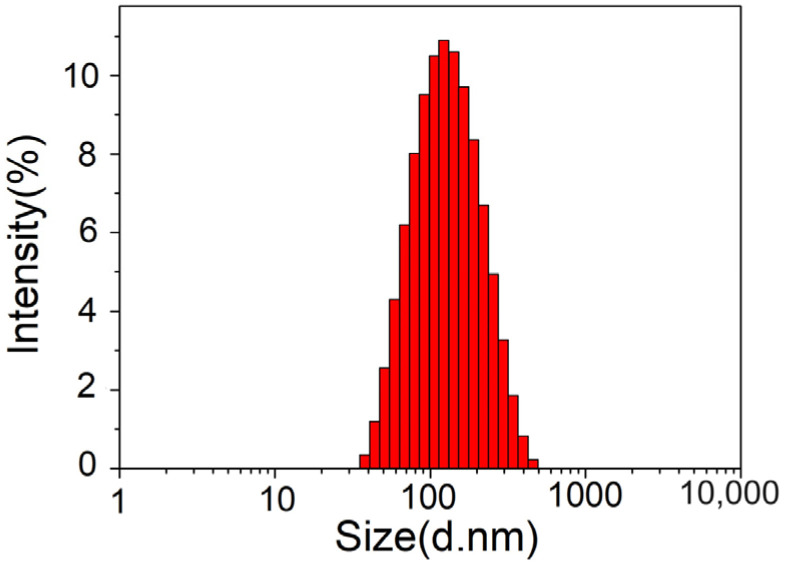
Particle size distribution of astaxanthin liposomes.

The average particle size, polydispersity index (PDI), and zeta potential are important parameters when evaluating liposomes. They reflect the properties and stability of samples, and also have important effects on the quality and efficacy exertion of liposomes. The samples were tested 3 times and averaged, and Figure 4 shows that the average particle size of an astaxanthin liposome was 105.8 ± 1.2 nm. The PDI of the astaxanthin liposomes was 0.238 ± 0.03, indicating a very good size distribution. It was previously reported that the particle size distribution is relatively uniform, and a sample has good stability when the PDI is less than 0.3 [18]. Therefore, the results showed that the astaxanthin liposome had good stability.

Zeta potentials reflect the magnitude of the repulsive force between charged particles; the more charges the nanoparticles carry, the more stable the nanoparticles are in the system. Mullar et al. [19] reported that the aggregation between nanoparticles can be effectively reduced so that their system was stabilized when the absolute value of the zeta potential was higher than or equal to 30 mV. Our measurement result showed that the zeta potential of the astaxanthin liposome was −38.0 ± 1.7 mV and its absolute value was higher than 30, demonstrating that the prepared astaxanthin liposome system had good stability. In addition, the system as a whole was negatively charged; this could facilitate the transportation of the liposomes in the body.

### 2.4. Encapsulation Efficacy and Loading Capacity of Astaxanthin Liposomes

During the preparation process for astaxanthin liposomes, the input amount of astaxanthin affects not only the encapsulation efficacy (EE), but also the drug-loading capacity (LC) [20]. The EE and LC measurement results for the astaxanthin liposomes of this study are shown in Table 1. When the input amount of astaxanthin increased, the drug LC of the astaxanthin liposomes gradually increased, but the EE gradually decreased. When the input amount of astaxanthin was 0.1 mg, the maximum EE was 88.83%.

### 2.5. Antioxidant Activity

**Figure 5:**
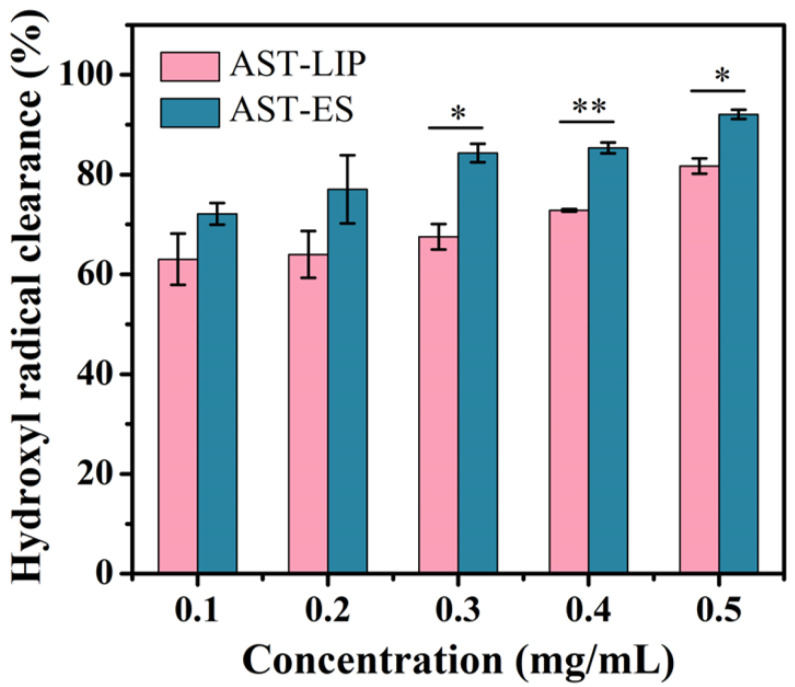
Hydroxyl radical scavenging capacity of astaxanthin liposomes in PBS (AST-LIP) and free astaxanthin molecules in ethanol solution (AST-ES). All data are shown as the mean ± SD; *n* = 3. * *p* < 0.05; ** *p* < 0.01.

As shown in Figure 5, the scavenging ability of the free astaxanthin molecules and astaxanthin liposomes to hydroxyl radicals improved with an increase in the astaxanthin concentration; however, the scavenging ability of the astaxanthin liposomes to hydroxyl radicals was lower than that of the free astaxanthin molecules. The reason for this phenomenon may be because the free astaxanthin molecules were encapsulated to form the astaxanthin liposomes, so the antioxidant activity of astaxanthin was limited to some degree. Nonetheless, as a type of sustained-release agent, the astaxanthin liposomes continuously exerted the biological activity of the astaxanthin molecules while maintaining their stability. When the astaxanthin concentration was lower than or equal to 0.2 mg/mL, there was no significant difference between the astaxanthin liposomes and the free astaxanthin molecules in scavenging hydroxyl radicals (*p* > 0.05). When the astaxanthin concentration was higher than 0.2 mg/mL, the clearance rates of the astaxanthin liposomes and astaxanthin molecules to hydroxyl radicals were significantly different (*p* < 0.05).

### 2.6. Stability Results of Astaxanthin Liposomes

**Figure 6:**
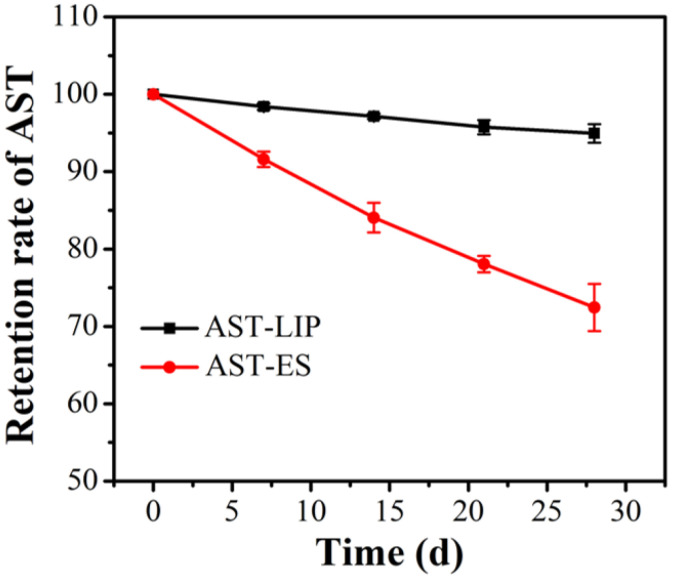
Storage stability of astaxanthin liposomes. All data are shown as the mean ± SD; *n* = 3.

In the process of sample storage, aggregation may occur between nanoliposomes due to the influence of external environment and other factors, which causes an increase in the nanoparticle size or even precipitation. Nanoliposomes can significantly improve drug stability and reduce drug leakage [21]. As shown in Figure 6, after being stored at 4 °C under dark conditions for four weeks, the astaxanthin content of the astaxanthin liposomes (AST-LIP) only decreased to (94.95 ± 0.85)% of the initial content, while the content of the free astaxanthin molecules in the ethanol solution (AST-ES) as the control group decreased to (72.45 ± 2.15)% of the initial content. Compared with free astaxanthin, the content of astaxanthin changed little after being embedded to form astaxanthin liposomes, indicating that the stability of astaxanthin significantly improved. The above results also demonstrated that the prepared astaxanthin liposomes could avoid the oxidation of astaxanthin and significantly improve the storage stability of astaxanthin.

## 3. Experimental Method

### 3.1. Materials

*Haematococcus pluvialis* with broken cell walls was bought from Xi’an Zebang Biotechnology Co., Ltd., Xian, China. Astaxanthin (standard sample) was purchased from Shanghai Yijing Industrial Co., Ltd., Shanghai, China. Cholesterol was bought from Tianjin Damao Chemical Instrument Supply Station, Tianjin, China. Soybean lecithin was purchased from Shenyang Tianfeng Bio-pharmaceutical Co., Ltd., Shenyang, China. Polyethylene glycol (−2000) was obtained from Tianjin Guangfu Fine Chemical Co., Ltd., Tianjin, China.

### 3.2. Establishment of the Astaxanthin Standard Curve

To measure the encapsulation efficiency of the astaxanthin liposomes, standard samples of astaxanthin were dissolved in ethanol at different concentrations (1, 2, 3, 4, and 5 μg/mL). The absorbance values of the above solutions were determined using an UV–vis spectrophotometer at a wavelength of 479 nm so that a standard curve of astaxanthin could be established.

### 3.3. Single-Factor Experiment

#### 3.3.1. Extraction Solvent Screening

Algal powder (0.1 g) was added into the mixed solvent (5 mL) containing ethyl acetate (EA) and ethanol (1:6, *v*/*v*), the mixed solvent (5 mL) containing chloroform and ethanol (6:1, *v*/*v*), and the mixed solvent (5 mL) containing dichloromethane and methanol (1:1, *v*/*v*), respectively. The above mixtures were digested in a constant-temperature water bath at 50 °C for 40 min, and then the supernatants were collected by centrifugation to measure the corresponding absorbance at a 479 nm wavelength. The extraction amount of astaxanthin was then calculated according to the astaxanthin standard curve. Therefore, the optimal extraction solvent could be confirmed.

#### 3.3.2. Extraction Time Screening

To confirm the optimal extraction time, algal powder (0.1 g) was added into the above optimal extraction solvent (5 mL) and digested in a constant-temperature water bath at 50 °C for 40, 50, 60, 70, and 80 min, respectively. Likewise, the supernatants were collected to measure the absorbance at a 479 nm wavelength, and the corresponding extraction amount of astaxanthin was calculated so that the optimal extraction time could be confirmed.

#### 3.3.3. Extraction Temperature Screening

To confirm the optimal extraction temperature, algal powder (0.1 g) was added into the above optimal extraction solvent (5 mL) and digested in a constant-temperature water bath at 20, 30, 40, 50, and 60 °C for the above optimal extraction time, respectively. Likewise, the supernatants were collected to measure the absorbance at a 479 nm wavelength, and the corresponding extraction amount of astaxanthin was calculated so that the optimal extraction temperature could be confirmed.

#### 3.3.4. Extraction Solid–Liquid Ratio Screening

The extraction solid–liquid ratios were set as 1:25, 1:50, 1:100, 1:150, and 1:200 (g/mL), respectively. Algal powder (0.1 g) was digested by the above optimal extraction solvent at the above optimal temperature for the above optimal time. After digestion, the supernatant was collected to measure the absorbance at a 479 nm wavelength so that the optimal extraction solid–liquid ratio could be confirmed.

#### 3.3.5. Extraction Time Screening

The above optimal factors were fixed, the algal powder (0.1 g) was digested by the extraction solvent five times, and the supernatant was collected to measure the absorbance at a 479 nm wavelength. According to the astaxanthin standard curve, the extraction amount of astaxanthin each time was calculated so that the optimal extraction times could be confirmed.

### 3.4. Preparation of Astaxanthin Liposomes

The preparation method referred to that of Gao et al. [22], rectified to some extent. First, the extracted astaxanthin (10 mg), cholesterol (6 mg), and soy lecithin (50 mg) were dissolved in anhydrous ethanol (5 mL). Then, the above mixture was gently heated and stirred until completely dissolved, and was then used as the lipid phase. A PBS solution (500 μL, 0.2 M, and pH 6.5) was mixed with 9500 μL deionized water, and then 10 μL Tween-80 and 5 mg PEG-2000 were added into the above mixed solution and heated to 45 °C to be used as the aqueous phase. The main role of Tween was to enhance the homogeneity, dispersibility, and stability between nanoliposomes, and PEG was used to improve the surface hydrophilicity and surface stability of the nanoliposomes. The lipid phase was added into the aqueous phase dropwise during stirring, and the mixture was then continually stirred for one hour until the anhydrous ethanol was completely volatilized. Finally, the astaxanthin liposome solution was freeze-dried to obtain a corresponding lyophilized powder. 

### 3.5. Characterization of Astaxanthin Liposomes

#### 3.5.1. Morphology Characterization

First, the astaxanthin liposome lyophilized powder dissolved in phosphate buffer saline (PBS) was negatively stained using phosphotungstic acid (0.0204 g/mL; pH = 7.0). The volume ratio of the astaxanthin liposome solution to phosphotungstic acid was 3:1. The negatively stained astaxanthin liposomes were dropped onto a carbon film and then the morphology and particle size of the sample were observed using a transmission electron microscope under a 100 kV voltage.

#### 3.5.2. Particle Size and Zeta Potential Characterization

The particle size of the astaxanthin liposomes was measured using dynamic light scattering (DLS) at 25 °C with a 90° light scattering angle. The surface zeta potentials of the astaxanthin liposomes were checked using a Malvern Zetasizer Nano-ZS90 (Malvern Instruments, Malvern, UK). The concentration of the samples used in the above characterization was 0.2 mg/mL. 

#### 3.5.3. Encapsulation Efficiency and Loading Capacity of Astaxanthin Liposomes

The lyophilized powder of the astaxanthin liposome dissolved in deionized water was mixed with a 50% ethanol solution and stirred for half an hour. After that, an appropriate amount of dichloromethane solution was added into the above mixture to extract the released astaxanthin. After standing for layering, the organic layer was separated and then used to measure the absorbance value at a 479 nm wavelength with a spectrophotometer. According to the standard curve of astaxanthin, the encapsulation efficiency (EE) and loading capacity (LC) of the astaxanthin liposomes were calculated using the following formulae:

(1) $$Encapsulation\;efficiency\left(EE\right)=\frac{Mass\;of\;astaxanth\;in\;in\;astaxanthin\;liposomes}{Mass\;of\;feeding\;astaxanthin}\times 100\%$$

(2) $$Loading\;capacity\left(LC\right)=\frac{Mass\;of\;astaxanth\;in\;in\;astaxanthin\;liposomes}{Mass\;of\;astaxanthin\;liposomes}\times 100\%$$

### 3.6. Antioxidant Activity (Measurement of Hydroxyl Radical Clearance Rate)

The antioxidant activity of astaxanthin referred to the report in [23], modified to some extent. An astaxanthin ethanol solution (free astaxanthin molecules dissolved in ethanol) and an astaxanthin liposome solution (astaxanthin liposomes suspended in PBS) at concentrations of 0.1, 0.2, 0.3, 0.4, and 0.5 mg/mL were prepared, respectively. A ferrous sulfate solution (0.5 mL; 5 mM), salicylic acid ethanol solution (0.5 mL; 5 mM), and H_2_O_2_ solution (0.5 mL; 3 mM) were added into the above prepared solutions (0.5 mL) in turn. After stirring the reaction for 30 min, the absorbance values of the mixed solutions were measured at a 510 nm wavelength and the absorbance values were set as A_x_. Deionized water was used as a substitute for the above astaxanthin ethanol and astaxanthin liposome solutions and the above operational process was repeated; the obtained absorbance values were set as A_0_.

Likewise, an astaxanthin ethanol solution and an astaxanthin liposome solution at concentrations of 0.1, 0.2, 0.3, 0.4, and 0.5 mg/mL were prepared, respectively. A ferrous sulfate solution (0.5 mL; 5 mM) and salicylic acid ethanol solution (0.5 mL; 5 mM) were added into the above prepared solutions (0.5 mL), respectively. After stirring the reaction for 30 min, the absorbance values of the mixed solutions were measured at a 510 nm wavelength and the absorbance values were set as A_y_.

Three-time parallel experiments were conducted for each concentration and the average absorbance value was obtained to calculate the corresponding clearance rate. The calculation formula was as follows:

Clearance rate = [A_0_ − (A_x_ − A_y_)]/A_0_ × 100% = 1 − (A_x_ − A_y_)/A_0_ × 100%

where A_0_ refers to the absorbance value of the deionized water added to FeSO_4_, salicylic acid, and H_2_O_2_; A_x_ refers to the absorbance value of the extraction solution added to FeSO_4_, salicylic acid, and H_2_O_2_; and A_y_ refers to the absorbance value of the extraction solution added to FeSO_4_ and salicylic acid, but without H_2_O_2_.

### 3.7. Stability of Astaxanthin Liposomes

The prepared astaxanthin liposome solution was sealed away from light and stored at 4 °C for 4 weeks, and a small amount of the above solution was taken every 7 days to determine the astaxanthin content. The astaxanthin ethanol solution with the same astaxanthin content served as the control group. By analyzing the astaxanthin content change, the stability of the astaxanthin liposome was investigated.

## 4. Conclusions

In this study, the influence of the type of extraction solvent, the extraction time and temperature, the solid–liquid ratio, and the extraction times on the extraction efficacy of astaxanthin from *Haematococcus pluvialis* was investigated via a single-factor experiment, and the optimal extraction conditions were confirmed as follows. The optimal extraction solvent was the mixed solvent containing ethyl acetate and ethanol with a volume ratio of 1:6; the optimal extraction time and temperature were 70 min and 60 °C, respectively; and the optimal solid–liquid ratio and extraction times were 1:100 (g/mL) and two times, respectively. In order to improve the stability of astaxanthin, astaxanthin liposomes were prepared by an ethanol injection using lecithin and cholesterol as carrier materials. The average particle size of the prepared astaxanthin liposomes was 105.8 ± 1.2 nm and the absolute value of the zeta potential was higher than 30, indicating the prepared astaxanthin liposomes had good dispersity. The antioxidant activity results showed that the hydroxyl radical scavenging rate of the astaxanthin liposomes was reduced to a certain extent compared with free astaxanthin molecules. However, the astaxanthin liposomes significantly improved the stability of astaxanthin and continually exerted its antioxidant activity. Therefore, the bioavailability and application of astaxanthin in biomedical or other fields may be significantly improved using an astaxanthin liposome strategy.

## Figures and Tables

**Table 1:** Effect of the input amount of astaxanthin on the EE and LC of liposomes.

Test Number	Astaxanthin Input (mg)	EE (%)	LC (%)
1	0.1	88.83	1.96
2	0.2	80.06	3.52
3	0.3	72.68	4.85
4	0.4	68.96	6.06
5	0.5	64.94	7.15

## Data Availability

No new data were created.
